# The composition of T cell subtypes in duodenal biopsies are altered in coeliac disease patients

**DOI:** 10.1371/journal.pone.0170270

**Published:** 2017-02-06

**Authors:** Janni V. Steenholt, Christian Nielsen, Leen Baudewijn, Anne Staal, Karina S. Rasmussen, Hardee J. Sabir, Torben Barington, Steffen Husby, Henrik Toft-Hansen

**Affiliations:** 1 Hans Christian Andersen Children’s Hospital, Odense University Hospital, Odense, Denmark; 2 Department of Clinical Immunology, Odense University Hospital, Odense, Denmark; Instituto Nacional de Ciencias Medicas y Nutricion Salvador Zubiran, MEXICO

## Abstract

One of the hallmarks of Celiac disease (CD) is intraepithelial lymphocytosis in the small intestine. Until now, investigations to characterize the T cell subpopulations within the epithelial layer have not discriminated between the heterodimeric co-receptor molecule, CD8αβ, and the possibly immunoregulatory CD8αα homodimer molecule. Besides TCRαβ+ CD4+ cells, no other phenotypes have been shown to be gluten-reactive. Using flow cytometry on lymphocytes from duodenal biopsies, we determined that the number of B cells (CD3- CD19+) and the number of CD3+ CD4- CD8- double-negative (DN) T cells were elevated 6–7 fold in children with CD. We next isolated and quantified intraepithelial lymphocytes (IELs) from biopsies obtained from patients (both children and adults) with CD, potential CD and non-CD controls. Flow cytometric analysis of the duodenal T cell subpopulations was performed including the markers TCRαβ, TCRγδ, CD4, CD8α and CD8β. Proportions of γδ T cells and CD8αβ^+^ cells among IELs were increased in CD patients, whereas proportions of CD4+ CD8αα+ and CD4+ single-positive T cells were decreased. Additionally, two gluten-reactive T cell lines (TCLs) derived from CD biopsies were analyzed for changes in proportions of T cell subsets before and after gluten stimulation. In a proliferation assay, dividing cells were tracked with carboxyfluorescein succinimidyl ester (CFSE), and both αβ and γδ T cells proliferated in response to gluten. Changes in duodenal T cell subpopulations in potential CD patients followed the same pattern as for CD patients, but with less pronounced effect.

## Introduction

Celiac disease (CD) is an immune-mediated disease that can develop in genetically predisposed individuals following ingestion of gluten [1]. Gluten-dependent small intestinal epithelial damage as well as presence of CD-specific antibodies in serum characterize the disorder [2]. The severity of epithelial affection may be graded in accordance with the Marsh classification [3], which in Oberhüber’s modification [4] ranges from grade 1 to 3(a-c) based on the level of intraepithelial lymphocytosis, crypt hyperplasia and villous atrophy. CD is estimated to affect about 1% of the population in western countries and appears to increase in prevalence [5–7]. About 95% percent of CD patients have the class II human leukocyte antigen (HLA)-DQ2 [8]. Most of the remaining patients have either HLA-DQ8 or the α or β-subunit of the DQ2 molecule [9, 10]. These antigen-presenting molecules have high affinity for deamidated gluten peptides (DGP). The deamidation is caused by the enzyme tissue transglutaminase (tTG), which converts neutral glutamines into negatively charged glutamic acids [11]. Cells with ability to present antigen on HLA class II molecules such as dendritic cells, macrophages, and possibly B cells, present DGP to CD4+ T cells in the lamina propria (LP), activating them and causing an inflammatory response to gluten, and eventually also leading to a destruction of epithelial cells by cytotoxic T cells [8, 12].

Intraepithelial lymphocytosis and the phenotypes and role of the intraepithelial lymphocytes (IELs) involved in the pathogenesis of CD are topics of great interest [13, 14]. The importance of gluten-reactive CD4^+^ Th1 cells have been appreciated for decades [15], but these cells are thought to primarily be present in the lamina propria, hence in another anatomical location than the IELs used by pathologists to diagnose the disease. Previously it has been demonstrated that both treated as well as untreated CD patients have a low level of possibly immunoregulatory CD4 CD8 double-positive T cells in the small intestinal epithelium [16]. Earlier studies found a fraction of the CD3^+^ intraepithelial lymphocytes (IELs), which could not be identified as either CD4^+^ or CD8^+^ in both CD patients with active disease as well as in treated patients [17–19]. These CD3^+^ CD4^-^ CD8^-^ cells might be γδ T cells, detection of which can be used to support histological CD diagnosis [20]. The fraction of γδ T cells has similarly been found elevated in CD patients with the increase persisting after years of gluten-free diet [21–23]. Likewise, dietary gluten can activate γδ T cells in the peripheral blood of CD patients, probably in an antigen-driven way [24]. The role in the pathogenesis of CD of these IELs has not been established and warrants further investigation.

The conventional CD8αβ co-receptor is an essential element in the TCR:MHC class I-interaction to present antigen to the CD8 effector T cell. CD8 T cells can also express a homodimer consisting of two α-chains [25], and T lymphocytes expressing two CD8α chains without the CD8β chain are called CD8αα. Conventional CD8 and CD4 T cells are TCR αβ^+^ T cells that were positively selected in the thymus. Both TCRαβ and TCRγδ T cells can express CD8αα only [26]. The function of the CD8αα is not yet fully understood, and it may not function as a co-receptor [27], also on CD4^+^ T cells. Evidence implies that the CD8αα molecule sequesters specific molecules required for the downstream transfer of the TCR-signal [14]. Consequently, the CD8αα-molecule indirectly represses the TCR-signal, thereby increasing the threshold for activation through the TCR. This mechanism is thought to be an immunoregulatory mechanism specific for the gut lining, where activation of pro-inflammatory IELs can impair the epithelial integrity, which is essential for protection against microorganisms.

In order to investigate potentially interesting lymphocyte subtypes in the pathogenesis of CD, we used flow cytometry to characterize B and T cell populations in duodenal biopsies from patients with CD and compared to controls. In this study, we particularly focused on the CD8αα-expression, or lack thereof, on TCRαβ cells, and on the phenotypes of γδ T cells. Furthermore, *in vitro* cultured T cell lines (TCLs) showing reactivity towards gluten were investigated as to which of the specified T cell subtypes proliferated after gluten-stimulation.

## Materials and methods

### 2.1. Subjects

Biopsies were obtained from patients undergoing diagnostic upper endoscopy at the Hans Christian Andersen’s Children Hospital or at the Department of Gastroenterology, Odense University Hospital, Denmark. Subject characteristics are listed in Table 1, and clinical characteristics of patients with celiac disease and disease controls in Table 2. All subjects had been instructed to maintain a diet including gluten-containing products at least 6 weeks before their diagnostic gastroscopy. To confirm or exclude a diagnosis of CD, a pathologist evaluated three or four duodenal biopsies and at least one biopsy from the duodenal bulb from each enrolled patient. Blood samples were obtained at the same time as the biopsies. All patients were tested for the presence of CD-associated haplotypes HLA-DQ2 or HLA-DQ8 using either sequence-specific-primer technique (Celiac Gene Alleles, Biodiagene) or microarray technique (EuroArray HLA-DQ2/DQ8, EuroImmun). For culturing of TCLs, only HLA-DQ2^+^ patients were included to fit the tissue type of the antigen-presenting HLA-DQ2^+^ B cells used in later proliferation assays.

**Table 1:** Subject characteristics from the analysis of duodenal biopsies.

Patient/Sex	Age Years	SerologyAnti-tTG titres^a^	HLA status DQ2/DQ8	IgA	Histology	Diagnose
**E11/M**	10	NEG	DQ8	Normal	Normal	Non-CD
**E12/F**	9	NEG	-/-	Normal	Normal	Non-CD
**E19/M**	11	NEG	DQ2/DQ8	Normal	Normal	Non-CD
**E21/F**	8	NEG	-/-	Normal	Normal	Non-CD
**E32/F**	64	NEG	-/-	Normal	Normal	Non-CD
**E33/M**	19	NEG	-/-	High	Normal	Non-CD
**E34/F**	62	NEG	DQ8	Normal	Marsh 1	Non-CD
**E37/F**	23	NEG	DQ2	Normal	Normal	Non-CD
**E39/F**	35	NEG	DQ2/DQ8	Normal	Normal	Non-CD
**E43/M**	21	POS/35	-/-	High	Normal	Non-CD
**E13/M**	13	POS/22	DQ2/DQ8	Normal	Normal	Potential CD
**E16/F**	14	POS/84	DQ2/DQ8	Normal	Marsh 1	Potential CD
**E26/F**	9	POS/78	DQ2/DQ8	Normal	Normal	Potential CD
**E36/F**	59	POS/53	DQ2	Normal	Marsh 1	Potential CD
**E14/M**	9	POS/116	DQ2	Normal	Marsh 3	CD
**E15/M**	12	POS/>150	DQ2	Normal	Marsh 3	CD
**E18/F**	9	POS/34	DQ2/DQ8	Normal	Marsh 3	CD
**E20/F**	7	POS/78	DQ8	Normal	Marsh 3	CD
**E22/F**	15	POS/98	DQ2	Normal	Marsh 3	CD
**E31/F**	16	POS/143	DQ2	Normal	Marsh 3	CD
**E38/F**	28	POS/>150	-/- (Carrier of the β-subunit of DQ2.5)	Normal	Marsh 3	CD
**E41/F**	21	POS/>150	DQ2	Normal	Marsh 3	CD
**E42/F**	17	POS/73	DQ2	Normal	Marsh 3	CD
**E45/F**	26	POS/>150	DQ8	Normal	Marsh 3	CD
**E46/F**	22	POS/54	DQ2	Normal	Marsh 3	CD

Table 1 shows the characteristics of the 11 CD patients, 4 potential CD patients, and 10 non-CD disease controls enrolled in the study.

^a^Cut-off value was 20 for a positive result; maximum value was 150.

**Table 2:** Clinical characteristics of patients with celiac disease and disease controls.

	Children (no, m/f, age range)	Adults (no, m/f, age range)
Celiac disease*	6, 2/4, 7–15 y	7, 0/7, 16–62 y
Disease controls	5, 3/2, 8–13 y	5, 1/ 4, 19–64 y

*including patients with potential celiac disease

Patients for this study were defined as having CD if they fulfilled two criteria: 1) a biopsy-verified Marsh II or III lesion, and 2) having the tissue-type HLA-DQ2, DQ8, or carrying one of the chains of the HLA-DQ2 heterodimer, as the vast majority of DQ2/8 negative CD patients carry either the α or β subunit of HLA-DQ2 [9]. Potential CD was defined as a subject with a positive titer of anti-tTG and carrying at least one of the predisposing HLA gene variants, but with either no histological lesions (Marsh 0) or only Marsh 1 lesions (intraepithelial lymphocytosis) [28]. Non-CD controls (children and adults) underwent an upper endoscopy due to suspicion of upper gastrointestinal pathology, for the children the final diagnoses were gastroesophageal reflux disease, recurrent abdominal pain, for the adults chronic diarrhea, dyspepsia, irritable bowel syndrome. All had normal findings at endoscopy, specifically with no CD-associated histopathology (not higher than Marsh 1), furthermore, they were required to have negative titers of anti-tTG antibodies in serum.

### 2.2. T cell, B cell and NK cell (TBNK) analysis

Lymphocytes were isolated for quantitative flow cytometry. Biopsies were homogenized by pressing them gently against a 70 μm mesh, lymphocytes were isolated by density gradient centrifugation using lymphoprep (Axis-Shield, Oslo, Norway) and analyzed using the BD Multi-test 6-color TBNK Reagent (BD Biosciences, San Jose, CA, USA) based on expression of CD3, CD16/56, CD45, CD19, CD4, and CD8α according to the manufacturer’s instructions on a BD FACSCanto II flow cytometer.

### 2.3. T cell lines

Generation of TCLs essentially followed our previously published protocol [29]. One–three intact biopsies were incubated overnight with 0.2 mg/ml of chymotrypsine-treated gluten peptides (CT-GLU) in RPMI 1640 Glutamax containing 10% human AB serum, penicillin/streptomycin and 2-mercaptoethanol (10% HS/MEPS). On day 1, biopsies were gently squeezed through a 40 μm mesh using a soft plunger and incubated for three days with 1x10^6^ cells/ml irradiated (50 Gy) autologous peripheral blood mononuclear cells (PBMCs) and 10% HS/MEPS containing 10 U/ml interleukin (IL)-2 and 1 ng/ml IL-15. Fresh medium containing IL-2 and IL-15 was added on day 4 and 5, and on day 7 TCLs received irradiated, allogenic PBMCs and 10 U/ml PHA to stimulate all surviving cells. TCLs were stimulated with IL-2 and IL-15 from day 9–13 and frozen when they had stopped proliferating after receiving 10% HS/MEPS without cytokines after day 13 (usually two or three days).

### 2.4. T cell proliferation assay

Gluten-reactivity of the TCLs was tested in a proliferation assay. Irradiated (75 Gy) immortalised B cells (VAVY cell line from International Histocompatibility Working Group, Seattle, WA, USA) were used as HLA-DQ2^+^ antigen-presenting cells (APCs) and were incubated with 0.1mg/ml deamidated (transglutaminase-treated) gluten (TG-GLU) in 96-well plates overnight. The following day, T cells were added to the wells in a T cell to APC ratio of 1:1.5. On day 3, ^3^H-thymidine was added to each well in a final concentration of 1 μCi and incubated for 16–18 hrs before harvesting of cells and measuring incorporated radioactivity on a beta-counter. Stimulation index (SI) was calculated as mean counts per minute (CPM) in presence of (TG-GLU) divided by mean CPM in the absence of antigen.

### 2.5. Carboxyfluorescein succinimidyl ester (CFSE) proliferation assay

Identification of proliferating T cell subtypes after gluten stimulation was performed by labelling TCLs with CellTrace (Life Technologies, Carlsbad, CA, USA), a highly permeable fluorescent agent, which is converted intracellularly to non-lipophilic carboxyfluorescein succinimidyl esters (CFSE). For every cell division the amount of intracellular covalently bound CFSE will be divided evenly between daughter cells [30]. T cells were re-suspended to 1x10^6^ cells per ml in preheated phosphate buffered saline (PBS) with 0.1% bovine serum albumin (BSA). Cells were then incubated with 2μl CellTrace solution per 2.5ml at 37° for 10 min. The process was stopped by adding 5x5ml of ice cold 10% HS/MEPS and incubation on ice for 5 min. Cells were then washed twice to eliminate any excess CellTrace or unbound CFSE. CellTrace proliferation assay had the same setup and length as the thymidine assay and CFSE-labelled T cells were added to the 96-wells in the same effector T cell:APC relationship as mentioned earlier. For comparison of subtype fractions before and after gluten stimulation, T cell phenotypes were analyzed prior to setup of proliferation assays. A thymidine assay was always conducted in parallel to determine the SI at the time of flow cytometric analysis.

### 2.6. Preparation of lamina propria and intraepithelial lymphocytes for flow cytometry

Biopsies were transported on ice in isotonic saline water directly from the endoscopy unit to the lab, and were immediately incubated in 1mM dithiothreitol (DTT) at 37°C for 20 min to dissolve surface mucus followed by separation of IELs and lamina propria lymphocytes (LPLs). Supernatant with possibly released IELs were sieved into a tube and stored on ice. Biopsies were then incubated in (0.5M) ethylenediaminetetraacetic acid (EDTA) at 37°C for 1 h to release IELs. After sieving the supernatant into the tube containing IELs, the remains of the biopsies were incubated in collagenase, 0.5mg/ml, at 37° for 30 min. Supernatants were sieved and released LPLs saved on ice.

Intraepithelial location of the IEL population was confirmed by flow cytometry using the expression of the CD103 molecule, an integrin (αEβ7) present on the majority of IELs (>96%) [31] which constituted 97% of the CD3^+^ cells in our IEL population. Contamination by LPL B cells (CD19^+^) among IELs was lower than 1% (results not shown).

### 2.7. Flow cytometric phenotyping of T cell subsets

Prior to labelling with conjugated antibodies, samples were incubated with Fc-block (BD Bioscience) for 10 min at RT. The following antibodies were used for phenotyping: PE-Cy5 anti-TCRαβ (LifeTechnologies), BV421 anti-TCRδ (BD Bioscience), APC-H7 anti-CD4 (BD Bioscience), PE anti-CD8α (BioLegend) and PE-Cy7 anti-CD8β (eBioscience). Before labelling with antibodies, cells were fixated and permeabelized using CytoFix/CytoPerm (BD Bioscience) according to instructions provided by the manufacturer. Forward and side scatters defined a lymphocyte gate without cell doublets. The T cell population comprises TCRαβ^+^ and TCRγδ^+^ cells, and each TCR type was defined as fractions of the T cell gate.

### 2.8. Statistics

Statistical analyses were performed using GraphPad Prism 5 (GraphPad Software Inc., San Diego California, USA). Non-parametric test for analysis of variance between groups was done using Kruskal-Wallis one-way ANOVA with Dunnett’s modification to detect differences between non-CD controls and the CD or potential CD groups. Tests were considered significant when the p value was <0.05.

### 2.9. Ethics

Adult patients and parents of the young patients gave written informed consent to the project, which was approved by the Ethics Committee for Biomedical Research in The Region of Southern Denmark (Project-ID: S20110043).

## Results

### 3.1. B cells and CD4 and CD8 double negative T cell numbers are increased in celiac disease biopsies

**Fig 1:**
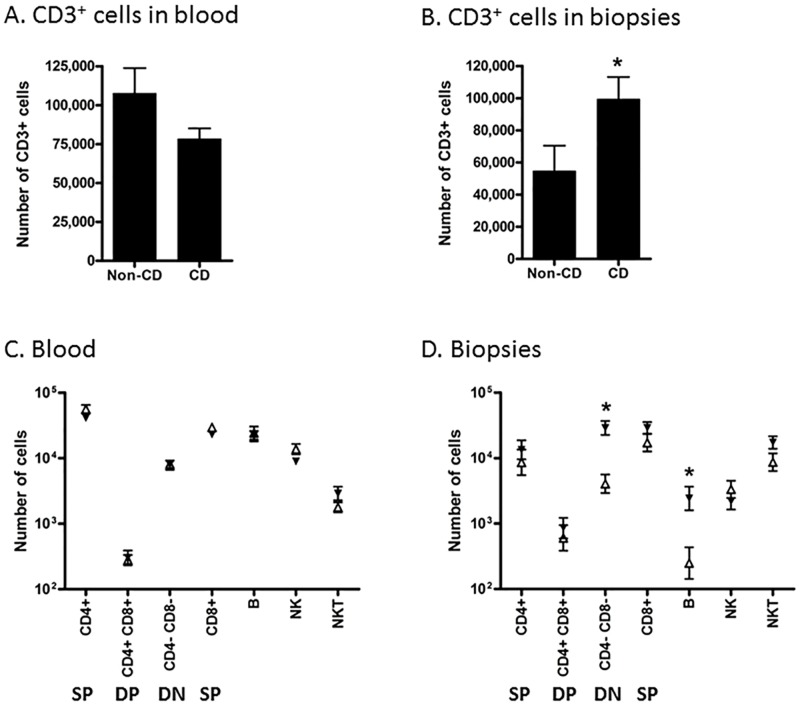
Flow cytometric quantification of lymphocyte populations in blood and duodenal biopsies. Panels A and B: average total number of CD3^+^ cells in 50 μl blood (A) and in one biopsy from each patient (B). Comparisons were made using a two-tailed unpaired Student’s t-test; error bars represent SEM; *: p<0.05; n = 13 (non-CD controls) and 14 (CD). Panels C and D: the same samples used in Panels A and B were analyzed for total number of subpopulations of lymphocytes in 50 μl blood (A) and in one biopsy (B) from each patient. Black triangles: CD patients (n = 14); open triangles: non-CD controls (n = 13). Comparisons between CD and control samples within each subpopulation were analyzed using a two-way ANOVA with Bonferroni post-tests; error bars represent SEM; *: p<0.05. Phenotypic markers of subpopulations: SP: CD3^+^ (CD4^+^ or CD8α^+^); DP: CD3^+^ CD4^+^ CD8α^+^; DN: CD3^+^ CD4^-^ CD8α^-^; B cells: CD3^-^ CD19^+^; NK cells: CD3^-^ CD16/56^+^; NKT cells: CD3^+^ CD16/56^+^.

In order to describe the lymphocytes that participate in CD pathogenesis, we measured the absolute numbers of lymphocyte subpopulations within each biopsy and a corresponding blood sample using quantitative flow cytometry with counting beads (Fig 1). There was no difference in the mean total number of CD3^+^ lymphocytes in blood samples between non-CD controls and CD patients (Fig 1A). In biopsies from non-CD controls, the mean total number of CD3+ lymphocytes was 54,700±15,790. This number was 82% higher (99,340 ±13,860 cells) in biopsies from CD patients (Fig 1B). There was no difference in numbers of lymphocyte subpopulations in blood samples from the patient groups (Fig 1C). The numbers of both CD3^+^ CD4^-^ CD8α^-^ double negative (DN) T cells as well as CD3^-^ CD19^+^ B cells were relatively higher in biopsies from CD patients compared to controls (Fig 1D). The number of DN cells was almost 6 fold higher (6,684±1,729 cells compared to 38,381±6,315 on average) corresponding to a difference of almost 32,000 cells per biopsy. The number of B cells was also almost 6 fold higher, but at a much lower level (1320±764 cells compared to 7709±3821 cells). The large difference in DN T cells can explain the majority (71%) of the higher number of total CD3^+^ lymphocytes in the biopsies from CD patients. There was no statistically significant difference in CD4^+^ or CD8α^+^ single positive (SP), CD4^+^ CD8α^+^ double positive (DP) CD3^+^ T lymphocytes, NK (CD16/56^+^, CD3^-^) or NKT (CD16/56^+^, CD3^+^) cells. In order to characterize subpopulations of T cells further, we performed another flow cytometric study with different biopsy donors, and included more specific phenotypic markers, particularly focusing on CD8α and β, as well as γδ T cells.

### 3.2. Proportions of TCR γδ T cells are upregulated in celiac disease

In total, 25 persons participated in this part of the study, of which 11 were confirmed to have CD, and four had potential CD, meaning positive anti-TG serology and HLA-DQ2/8 positive, but normal or only Marsh 1 histology (Table 1). The biopsy-derived cells were separated into intraepithelial cells (IELs) and lamina propria lymphocytes (LPLs), and analyzed by flow cytometry. We investigated if the CD4^-^ CD8α^-^ DN T cells, which were higher in the whole CD biopsies (Fig 1D), also were present in the intraepithelial layer, and further, if these cells were belonging to the TCRγδ or αβ T cell subsets.

**Fig 2:**
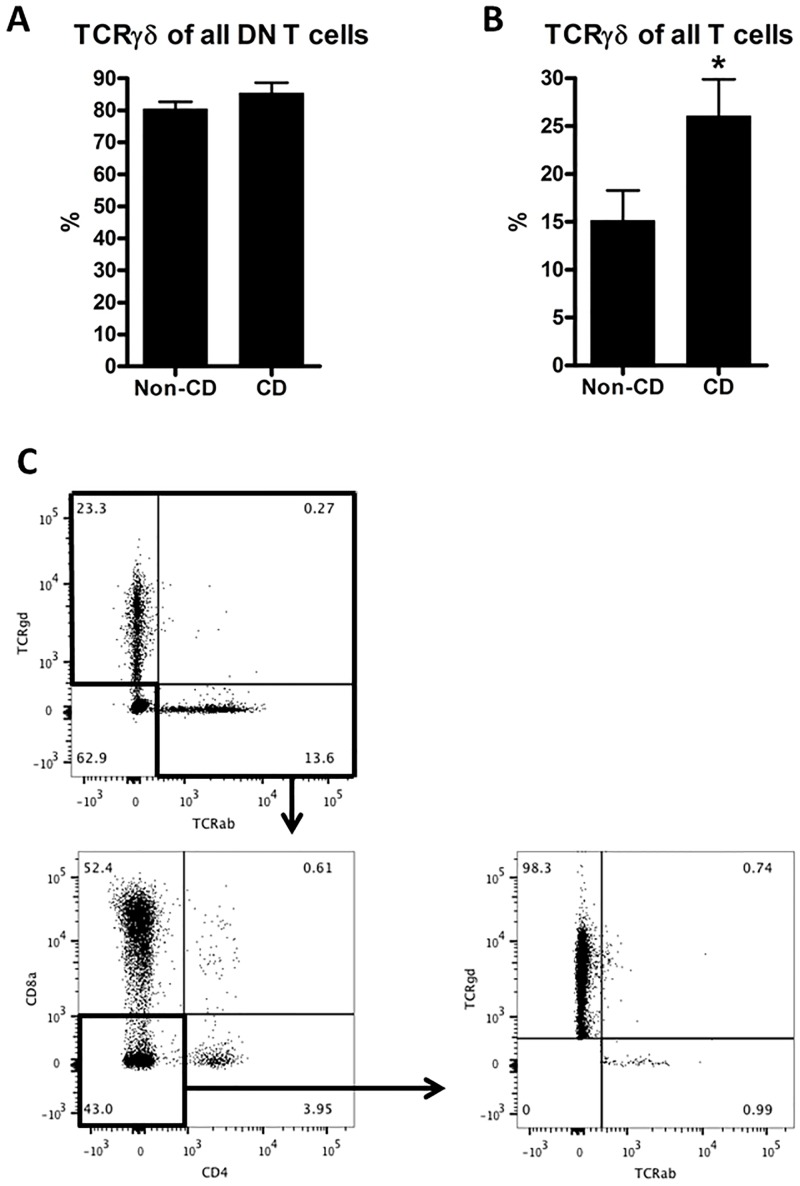
Flow cytometric analysis of intraepithelial lymphocytes. Proportions of TCRγδ^+^ cells among CD4^-^ CD8α^-^ (DN T cells) (A). Proportions of TCRγδ^+^ cells among all T cells (B). Gating strategy to identify TCRγδ^+^ DN T cells (C): first, all non-T cells are gated out based on lack of αβ or γδ TCR, next, CD4+ and CD8a^+^ cells are gated out, and the resulting population analyzed for expression of TCRδ and TCRαβ. Comparisons were made using a two-tailed unpaired Student’s t-test; error bars represent SEM; *: p<0.05; n = 10 (non-CD controls) and 11 (CD).

Indeed, in the IEL compartment, we could demonstrate that 80–85% of all DN T cells were TCRγδ^+^ in both CD patients and controls (Fig 2A). When we analyzed the proportion of γδ T cells among all IEL T cells, we found a 72% higher number of γδ T cells in CD biopsies (Fig 2B), indicating that γδ T cells are selectively recruited to the IEL compartment in CD biopsies.

### 3.3. The proportion of CD8αα^+^ cells is higher among TCRγδ than among TCRαβ cells

**Fig 3:**
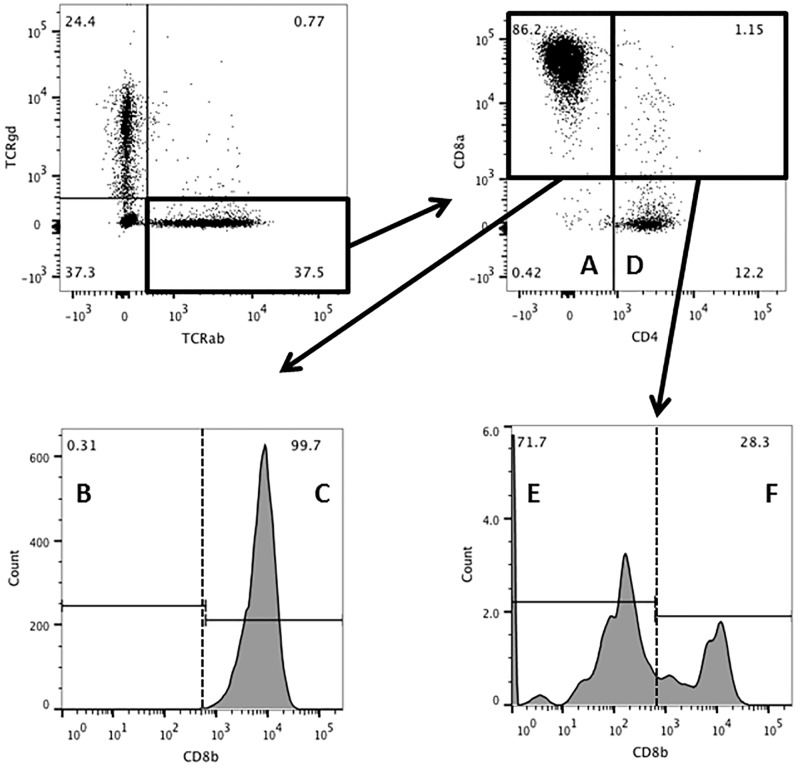
Gating strategy and definition of TCRαβ^+^ subpopulations. The upper left dotplot defines the lymphocytes as either TCRαβ or γδ positive. The upper right dotplot defines A: DN (CD4^-^ CD8α^-^) and D: CD4 (CD4^+^ CD8α^-^); the lower left histogram defines B: CD8αα (CD4^-^ CD8α^+^ CD8β^-^) and C: CD8 (CD4^-^ CD8α^+^ CD8β^+^); the lower right histogram defines E: CD4 CD8αα (CD4^+^ CD8α^+^ CD8β^-^); and F: DP (CD4^+^ CD8α^+^ CD8β^+^). TCRγδ^+^ cells were analyzed analogously.

**Fig 4:**
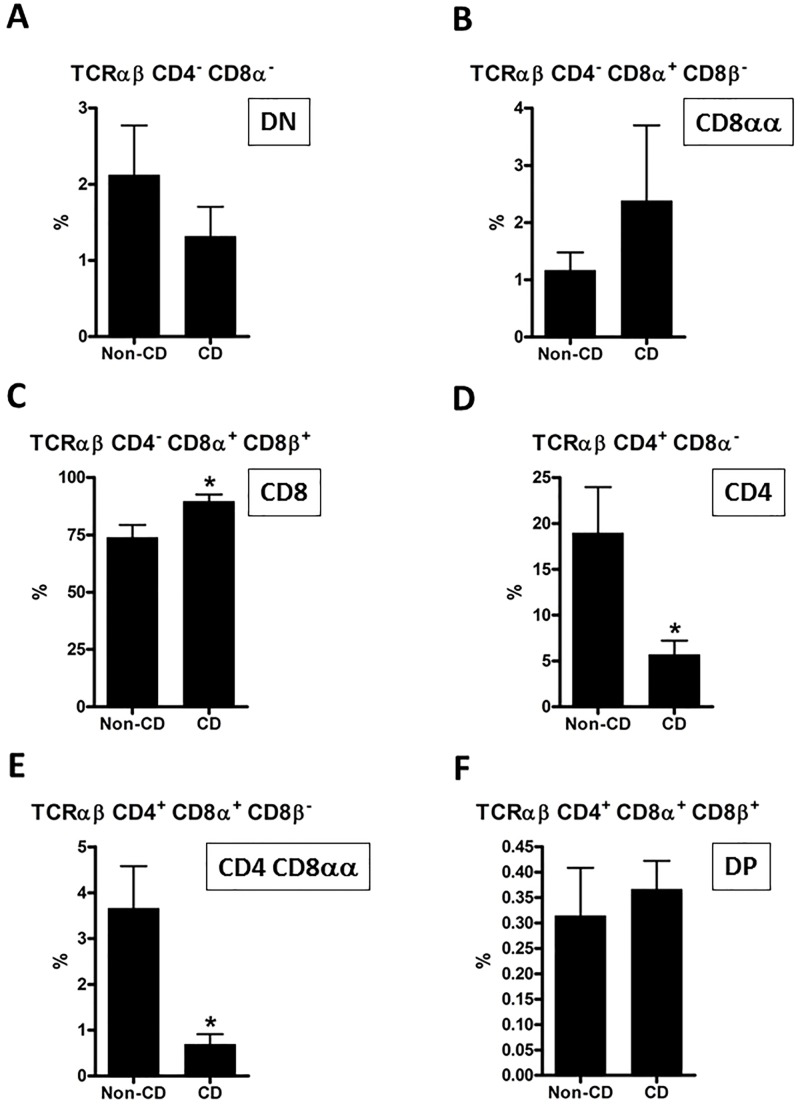
Flow cytometric analysis of intraepithelial TCRαβ subpopulations. Based on the gating and defined subpopulations illustrated in Fig 3, each subpopulation was analyzed separately to identify differences between CD and controls. Comparisons were made using a two-tailed unpaired Student’s t-test; error bars represent SEM; *: p<0.05; n = 10 (non-CD controls) and 11 (CD).

**Fig 5:**
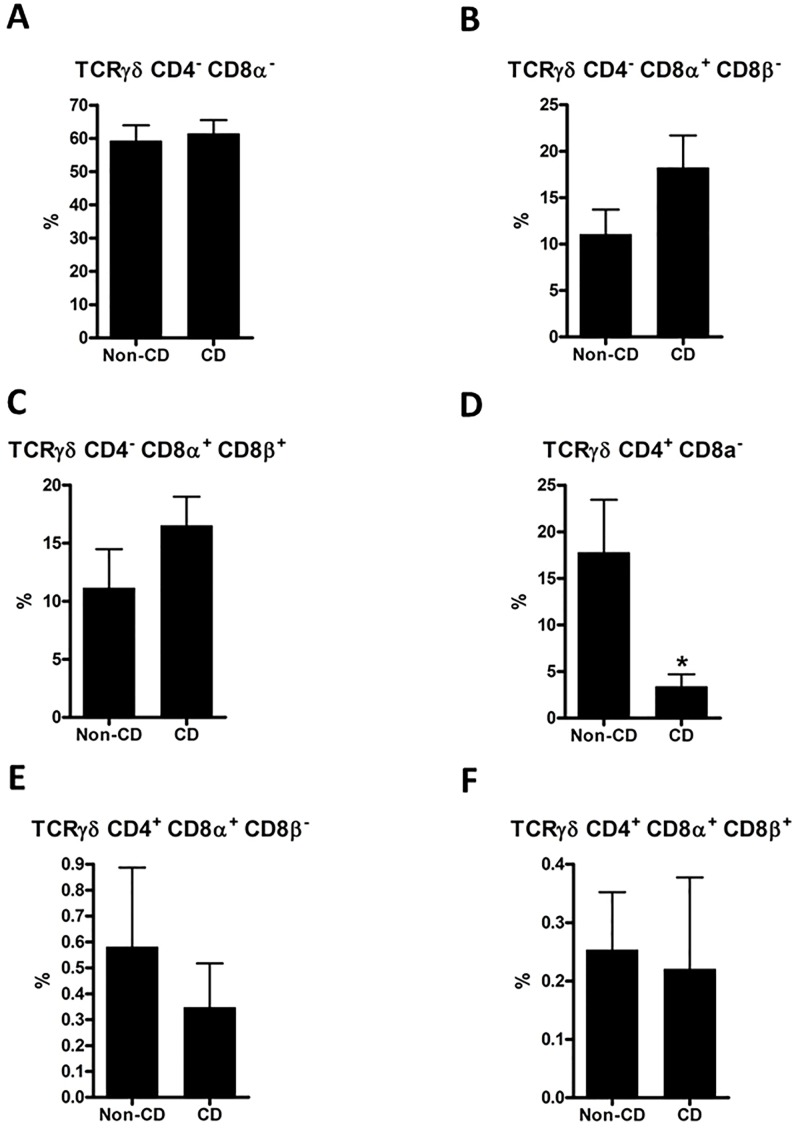
Flow cytometric analysis of intraepithelial TCRγδ subpopulations. TCRγδ^+^ subpopulations were analyzed in the same manner as TCRαβ subpopulations in Fig 4. Comparisons were made using a two-tailed unpaired Student’s t-test; error bars represent SEM; *: p<0.05; n = 10 (non-CD controls) and 11 (CD).

Phenotypic definitions of lymphocyte subpopulations are illustrated in Fig 3 with TCRαβ used as example; TCRγδ cells were analyzed analogously. In a comparison of IEL CD4^-^ CD8α^+^ CD8β^-^ (CD8αα^+^ cells), we found a striking difference between TCRαβ and TCRγδ cells. In both CD and non-CD controls samples, CD8αα^+^ constituted around 1–2% of the TCRαβ cells, whereas they constituted around 15% of the TCRγδ cells (Figs 4B and 5B). The conventional CD8^+^ cells (CD8αβ^+^) also constituted approx. 15% of TCRγδ cells with no difference between CD and non-CD controls (Fig 5C)

### 3.4. Proportions of IEL TCRαβ cell subpopulations are altered in CD patients

Among IEL TCRαβ cells, CD8αβ^+^ cells constituted about 74% in non-CD controls and were significantly elevated in CD patients to almost 90% (Fig 4C). Interestingly, the mean proportion of CD4^+^ CD8α^-^ cells was 19% in non-CD controls and only 6% in CD patients (Fig 4D). There was also a decrease in the proportion of TCRαβ CD4^+^ CD8αα^+^ cells from around 4 to 1% in CD patients (Fig 4E). Among the other three subpopulations identified, there were no significant differences between non-CD controls and CD patients (Fig 4A, 4B and 4F).

### 3.5. Analysis of lamina propria lymphocytes in CD patients and controls showed some differences and some similarities to IELs

**Fig 6:**
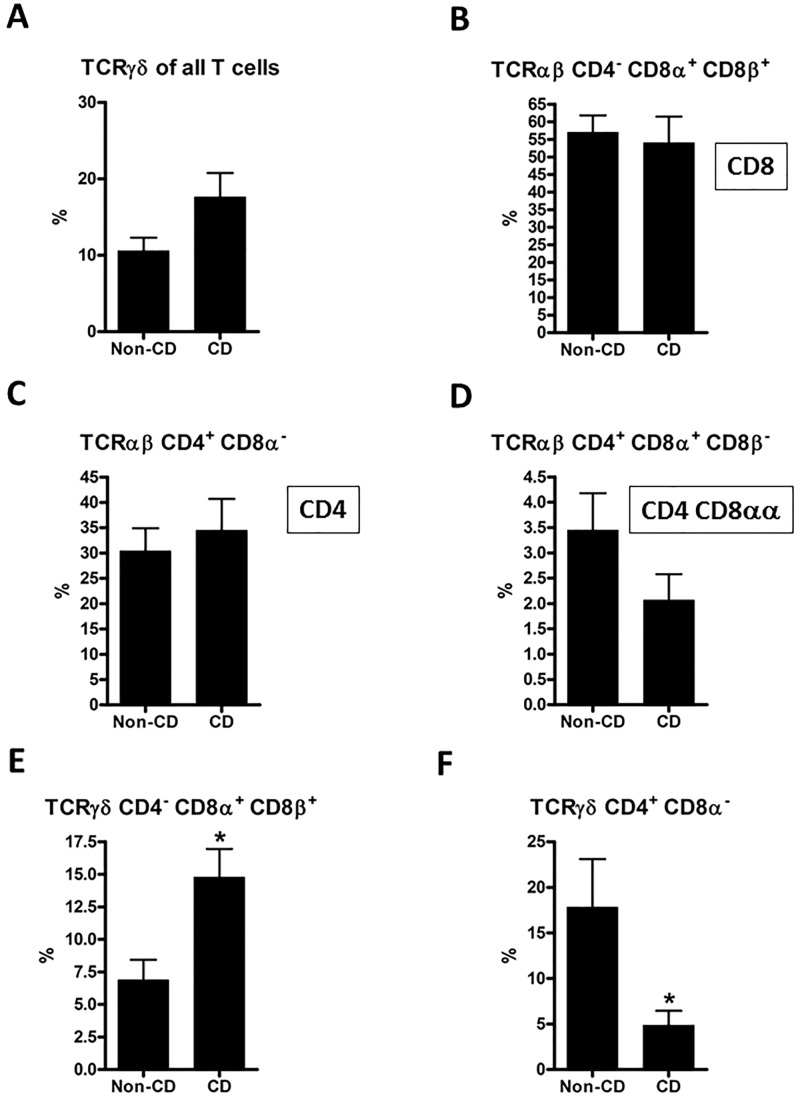
Flow cytometric analysis of lamina propria TCRαβ and TCRγδ lymphocytes. Subpopulations that differed significantly between CD patients and controls in the IEL, or differed significantly in the LPL compartment are presented here. Comparisons were made using a two-tailed unpaired Student’s t-test; error bars represent SEM; *: p<0.05; n = 10 (non-CD controls) and 11 (CD).

We performed an analogous analysis of the cells in the lamina propria compartment of the biopsies. Results from the subtypes that differed significantly between CD patients and controls in the IEL, or differed significantly in the LPL compartment, are presented in Fig 6. All other comparisons of LPL subtypes showed no significant differences between CD patients and controls (data not shown). TCRγδ cells showed the same trend in CD patients among LPLs (Fig 6A) as among IELs, where the increase reached statistical significance (Fig 2B). In contrast, no trend was observed among LPLs for CD8^+^ cells (Fig 6B), which were elevated among IELs (Fig 4C). The lower proportion of CD4 and CD4 CD8αα cells in CD patients found among IELs was not found among LPLs (Fig 6C and 6D). In the LPL compartment, TCRγδ CD4^-^ CD8a^+^ CD8b^+^ cells were significantly increased in CD patients in accordance with the trend among IELs. TCRγδ CD4^+^ CD8α^-^ constituted a significant lower proportion in CD patients both among IELs and LPLs.

### 3.6. Potential CD patients resemble CD patients more than non-CD controls

**Fig 7:**
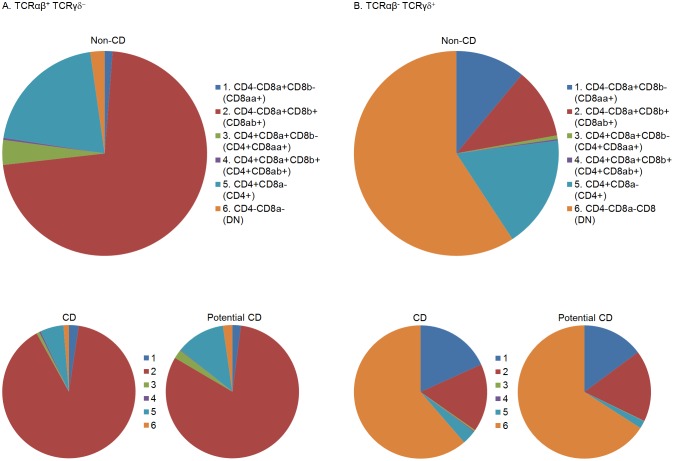
Illustration of the mean flow cytometric proportions of six identified intraepithelial T cell subpopulations. Three groups of subjects are presented: CD and non-CD as in Figs 2 and 4–6, as well as potential CD. Potential CD subjects had CD-associated HLA tissue types and positive anti-TG2 in serum, but no crypt hyperplasia or villous atrophy (Marsh grade 0 or 1). TCRαβ^+^ TCRγδ^-^ IELs are shown in Panel A, and TCRαβ^-^ TCRγδ^+^ IELs in Panel B. n = 10 (non-CD controls), 4 (potential CD) and 11 (CD).

Four patients fulfilled the criteria for potential CD. They had CD-associated HLA tissue types and positive anti-TG2 in serum (relatively low titres: 22–84 U), but no crypt hyperplasia or villous atrophy (Marsh grade 0 or 1) (Table 1), and were not recommended to follow a gluten-free diet. These four patients were excluded from our direct comparison of CD versus Non-CD controls. However, it is interesting to consider whether the flow cytometric characteristics of biopsies in the “potential CD” state resemble the normal state or the altered state of CD biopsies. We observed that the average pattern from potential CD patients showed an intermediate pattern for TCRαβ cells, and for TCRγδ cells resembled the pattern from CD patients more than non-CD controls (Fig 7).

### 3.7. Both αβ T cells and γδ T cells in gluten-reactive T cell lines proliferate in response to gluten

Two gluten-reactive TCLs from two CD patients were stimulated with gluten to determine which T cell subpopulations would proliferate. A phenotypic characterization of the cell lines was performed prior to gluten stimulation (GS) and compared to contents of subtypes after GS. Baseline compositions of the TCLs were different in proportions, but were mainly composed of the same four phenotypes: For conventional TCRαβ cells: CD8αβ and CD4^+^ cells; and for TCRγδ cells: either the DN phenotype or a CD8 SP phenotype. To determine if both αβ T cells and γδ T cells proliferate in response to gluten and possibly contributed to the previously observed changes in proportions in CD biopsies, the TCL1 and the TCL2 was CFSE-labeled prior to GS. Altogether, 10.7% of T lymphocytes had proliferated in TCL1 in response to gluten, and for TCL2, 19.2% had proliferated in response to gluten. Proliferating cells were divided on the basis of their TCR type. In TCL1, 7.38% of TCRαβ and 34.2% of TCRγδ cells had proliferated. In TCL2, the percentages were 18% for TCRαβ cells and 21.9% for TCRγδ cells.

## Discussion

In the first round of experiments, we quantified lymphocyte populations in blood samples and duodenal biopsies from patients with CD (previously un-diagnosed) and compared to biopsies from non-CD subjects. We found that the number of B cells and the number of DN T cells were significantly higher (6–7 fold) in patients with CD. In a second round of experiments, we analyzed IELs and LPLs separately using an extended panel of phenotypic T cell markers. As expected, the previously observed increase in number of DN T cells reflected an increase in γδ T cells. We further showed that IEL T cell subtypes were altered in biopsies from CD patients, and that potential CD patients showed a pattern more similar to CD than to non-CD controls. Finally, both αβ T cells and γδ T cells in gluten-reactive TCLs were able to proliferate in response to gluten.

The relatively higher number of B cells is likely different from the previously reported increase in plasma cells, since we measured CD19^+^ cells. The higher number of CD19^+^ B cells may reflect a role of these cells in CD pathology as previously discussed [12], possibly as antigen-presenting cells. By sub-classifying T cells using the CD8β-specific antibody in combination with TCR-specific antibodies, we have presented a detailed picture of T cell subpopulations in the lamina propria and intraepithelial compartments. We found TCRαβ^+^CD8^+^ SP T cells (conventional cytotoxic T cells) increased in proportions in CD patients as compared to non-CD patients. This subset is thought to be the main contributors to the epithelial damage observed in the duodenal mucosa of CD patients [32, 33].

TCRγδ+ cells are considered unconventional, and their function is supposedly in between the adaptive and the innate immune system [14, 34]. It has long been known that numbers of γδ T cells is increased in CD mucosa, as we also confirmed by quantitative flow cytometry. As expected, we observed with CFSE labelling, that αβ T cells in the gluten-reactive T cell lines proliferated in response to gluten. Interestingly, even higher proportions of γδ T cells in the TCLs proliferated after the stimulation, suggesting that γδ T cells are directly responding to gluten antigens, although the proliferation could also be due to indirect stimulation by secreted cytokines from the gluten-specific αβ CD4 T cells responding to gluten. In any case, this emphasizes that gluten-induced activation of γδ T cells (directly or indirectly) in the gut mucosa play a potential role in the pathogenesis of CD, or, alternatively, be involved in repair [35].

An interesting observation in our study concerning the phenotypic characterization of IELs was the finding that the proportional changes of T cell subsets of the group of potential CD patients seemed to follow the pattern of CD patients rather than that of non-CD patients. Patients with potential CD do not have degenerated architecture of the epithelial mucosa, but they may present with the relative intraepithelial lymphocytosis, termed a Marsh grade 1. The existence of potential CD is debated [28, 36], but some of the symptomatic potential CD patients benefit from a gluten free diet, and asymptomatic potential CD patients appear to have a high risk of developing full-blown CD later in life [37–39]. Our results imply that potential CD in terms of lymphocyte composition in the duodenal epithelium is an intermediate state between normal and the diseased state with epithelial damage.

Experiments in mice have indicated that in the presence of excess interleukin (IL)-15, diet-antigen specific CD4^+^ IELs of the small intestine will obtain increased cytotoxic potential as a consequence of stimulation with their cognate antigen [40]. An almost simultaneous upregulation of the supposedly immunosuppressing CD8αα-homodimer was additionally induced on the CD4^+^ IELs. Increased levels of IL-15 has been detected in the small intestine of CD patients [41], leading to the hypothesis that the same mechanisms could apply to CD4 IELs in humans with gluten as the cognate diet-antigen. We found that proportions of IEL CD4 CD8αα T cells were decreased by 81% in CD biopsies. Whether this reflects a pathophysiologically relevant lack of immunosuppression by these cells in CD patients leading to enhanced inflammation remains to be established, as no studies of their cytotoxic or regulatory potential were performed in the present report. Not much is known about potential cytotoxic or protective CD4^+^ T cells residing within the IEL compartment of the small intestine, and this area warrants further investigation.
